# Laparoscopic detection and resection of occult liver tumors of multiple cancer types using real-time near-infrared fluorescence guidance

**DOI:** 10.1007/s00464-016-5007-6

**Published:** 2016-06-29

**Authors:** Leonora S. F. Boogerd, Henricus J. M. Handgraaf, Hwai-Ding Lam, Volkert A. L. Huurman, Arantza Farina-Sarasqueta, John V. Frangioni, Cornelis J. H. van de Velde, Andries E. Braat, Alexander L. Vahrmeijer

**Affiliations:** 1 Department of Surgery, Leiden University Medical Center, Albinusdreef 2, 2300 RC Leiden, The Netherlands; 2 Department of Pathology, Leiden University Medical Center, Leiden, The Netherlands; 3 Division of Hematology/Oncology, Department of Medicine, Beth Israel Deaconess Medical Center, Boston, MA USA; 4 Curadel, LLC, Worcester, MA USA; 5 Department of Radiology, Beth Israel Deaconess Medical Center, Boston, MA USA

**Keywords:** Tumor imaging, Surgical navigation, Intraoperative guidance, Indocyanine green, Hepatic metastases, Fluorescence imaging

## Abstract

**Background:**

Tumor recurrence after radical resection of hepatic tumors is not uncommon, suggesting that malignant lesions are missed during surgery. Intraoperative navigation using fluorescence guidance is an innovative technique enabling real-time identification of (sub)capsular liver tumors. The objective of the current study was to compare fluorescence imaging (FI) and conventional imaging modalities for laparoscopic detection of both primary and metastatic tumors in the liver.

**Methods:**

Patients undergoing laparoscopic resection of a malignant hepatic tumor were eligible for inclusion. Patients received standard of care, including preoperative CT and/or MRI. In addition, 10 mg indocyanine green was intravenously administered 1 day prior to surgery. After introduction of the laparoscope, inspection, FI, and laparoscopic ultrasonography (LUS) were performed. Histopathological examination of resected suspect tissue was considered the gold standard.

**Results:**

Twenty-two patients suspected of having hepatocellular carcinoma (*n* = 4), cholangiocarcinoma (*n* = 2) or liver metastases from colorectal carcinoma (*n* = 12), uveal melanoma (*n* = 2), and breast cancer (*n* = 2) were included. Two patients were excluded because their surgery was unexpectedly postponed several days. Twenty-six malignancies were resected in the remaining 20 patients. Sensitivity for various modalities was 80 % (CT), 84 % (MRI), 62 % (inspection), 86 % (LUS), and 92 % (FI), respectively. Three metastases (12 %) were identified solely by FI. All 26 malignancies could be detected by combining LUS and FI (100 % sensitivity).

**Conclusion:**

This study demonstrates added value of FI during laparoscopic resections of several hepatic tumors. Although larger series will be needed to confirm long-term patient outcome, the technology already aids the surgeon by providing real-time fluorescence guidance.

**Electronic supplementary material:**

The online version of this article (doi:10.1007/s00464-016-5007-6) contains supplementary material, which is available to authorized users.

Major advances have been achieved in the treatment of patients with liver-only metastases and primary liver tumors. Several studies and meta-analyses show improved overall survival after aggressive resection [1–3]. Recurrence-free survival, however, is rather low. Up to 69 % of patients with colorectal liver metastases develop local recurrence or new metastases, with the majority in the first year [4]. Similar rates are reported in patients undergoing resection of hepatocellular carcinoma (HCC) [5]. These numbers are not entirely explained by incomplete resections and support the hypothesis that small malignant lesions are missed during resection.

It is well known that preoperative imaging modalities, including computed tomography (CT) and magnetic resonance imaging (MRI), have relatively low sensitivity for sub-centimeter lesions due to their limited spatial resolution. Moreover, altered body position during surgery and time interval between preoperative imaging and surgery make translation of preoperative images challenging. Extensive intraoperative inspection and palpation of the liver is therefore of vital importance. Palpation, however, is impossible during minimally invasive procedures. Laparoscopic ultrasonography (LUS) can assist in detecting tumors, but acoustic reflection artifacts can obscure tumors in the first millimeters below the liver capsule [6]. In short, surgeons need a highly sensitive, real-time intraoperative imaging modality to identify small superficial tumors in the liver.

Ishizawa et al. [7] reported in 2009 that an intravenous injection of the safe and widely used dye indocyanine green (ICG) causes “bullseyes” of fluorescence to be formed around primary or metastatic tumors in the liver. Defective biliary clearance in the transition area between tumor and normal liver tissue and in primary liver tumors results in ICG retention, which can be visualized using a near-infrared (NIR) fluorescence imaging system. Our group showed that this method of detection is feasible during resection of hepatocellular carcinoma (HCC) and hepatic metastases of uveal melanoma (UMLM) and colorectal cancer (CRLM) [8–10]. The added value of fluorescence imaging during oncological liver surgery is twofold: (1) It can demarcate liver tumors and provide real-time resection margin assessment, and (2) it can identify otherwise undetectable, i.e., occult, (sub)capsular liver tumors. NIR fluorescence imaging can be especially valuable during minimally invasive procedures, because surgeons are deprived of tactile feedback. Based on previous study results, fluorescence imaging is now routinely performed during laparoscopic liver surgery in our hospital if patients provide informed consent. The purpose of the present study was to compare sensitivity and positive predictive values of fluorescence imaging with CT, MRI, visual inspection, and LUS during laparoscopic resection of several tumor types in the liver.

## Materials and methods

### Patients

The local Medical Ethics Committee and the Departments of Surgery and Anesthesiology approved the implementation of NIR fluorescence imaging during oncologic liver surgery as standard of care. Informed consent from all patients was obtained. ICG (25 mg vials, Pulsion Medical Systems, Munich, Germany) was dissolved in 10 mL sterile water to yield a 2.5 mg/L (3.2 mM) concentration. Subsequently, a bolus of 4 mL containing 10 mg ICG was administered intravenously 24 h prior to surgery. Dose and time of administration were optimized in our previous study [8].

All patients undergoing laparoscopic staging or resection of a liver tumor were eligible for inclusion. Exclusion criteria were eGFR <55, pregnancy, lactation, and an allergy to iodine, shellfish, or ICG. Preoperative imaging was performed using computed tomography (CT) with or without positron emission tomography (PET) and in most cases also using magnetic resonance imaging (MRI), all within 6 weeks prior to surgery.

### Near-infrared fluorescence imaging

Intraoperative imaging during surgery was performed using the laparoscopic high-definition fluorescence imaging system (Karl Storz GmbH & Co. KG, Tuttlingen, Germany). The system includes a light source for visible and 760 nm (ICG mode) light, a plasma light guide, and a 30°, 10 mm laparoscope containing optical filters. The system allows easy switching between white light and ICG modes using a foot pedal. Images were recorded using a charge-coupled camera device.

Ex vivo fluorescence imaging of resected tissue specimens was performed at the pathology department using the previously described fluorescence-assisted resection and exploration (FLARE^®^, Beth Israel Deaconess Medical Center, Boston, MA, USA) prototype surgical imaging system [11]. In addition, the location of ICG in resected tissue was assessed using fluorescence microscopy. Digital images were made using the Leica DM5500B digital microscope (Leica Microsystems B.V., Son, the Netherlands) equipped with a Leica DFC365FX camera and LAS X software (version 2.6.0) for image acquisition and processing.

### Procedure

The procedure started with the inspection of the abdominal cavity for the presence of extrahepatic metastases. When none were found, the liver capsule was inspected, followed by NIR fluorescence imaging. Subsequently, LUS was performed by a dedicated radiologist. Each suspect lesion detected by inspection, fluorescence imaging, and/or LUS was resected when the surgeon considered it to be safe and relevant.

All resected liver lesions were routinely analyzed at the pathology department, and the method of detection was correlated with histopathological findings. Histopathological diagnosis was considered the gold standard. Sensitivity and positive predictive value of every resected lesion was calculated for all imaging modalities. Only malignant lesions were considered true positives. Statistical analysis was performed using SPSS version 22.0 software (SPSS, ^©^IBM Corporation, Somer, NY, USA).

## Results

### Patient characteristics

Twenty-two patients planned to undergo laparoscopic staging (*n* = 4) or resection (*n* = 19) of one or multiple tumors confined to the liver were included from April 2013 to November 2015. Patient characteristics are shown in Table 1. One patient, with a suspected intrahepatic cholangiocarcinoma, was included twice. First, he underwent a diagnostic laparoscopy followed by resection of the liver tumor 2 months later.

**Table 1:** Patient characteristics

Total patients, *N*	22
Age at surgery, median [range]	65 [28–76]
Gender, % (*n*)	
Female	45 [5]
Male	55 [11]
BMI, median [range]	23.0 [18.8–36.6]
ASA classification, % (*n*)	
1	9 [16]
2	82 (18)
3	9 [16]
4	0 (0)
Liver cirrhosis, % (*n*)	14 [15]
Neoadjuvant therapy, % (*n*)	27 [30]

*Abbreviations* *ASA* American Society of Anesthesiologists

Preoperative working diagnosis differed from final histopathological diagnosis in four patients. One patient presented with a large, atypical mass in segments 2 and 3 was suspected to have (fibrolamellar) HCC by radiological imaging. Final diagnosis demonstrated focal nodular hyperplasia (FNH). The second patient, with a history of laparoscopic cholecystectomy, presented with abdominal pain and preoperative images showed a liver lesion suspicious for HCC. This lesion was found to be liver tissue herniated through one of the old trocar incisions. The third patient presented with a history of breast cancer and a FDG-PET-positive lesion in segment 4b, suspicious for metastasis. Instead, the final diagnosis was intrahepatic cholangiocarcinoma. The fourth patient was suspected to have an intrahepatic cholangiocarcinoma and simultaneous liver metastases of a non-small cell lung carcinoma (NSCLC). The large liver tumor suspicious for cholangiocarcinoma was not biopsied during the first procedure. Two other suspect lesions in the liver, either metastases of NSCLC or hepatic adenomas, were biopsied and diagnosed as hepatic adenomas. The large tumor was resected in a second procedure, and final histopathological evaluation indeed showed an intrahepatic cholangiocarcinoma.

### Pre- and intraoperative tumor detection

**Fig. 1:**
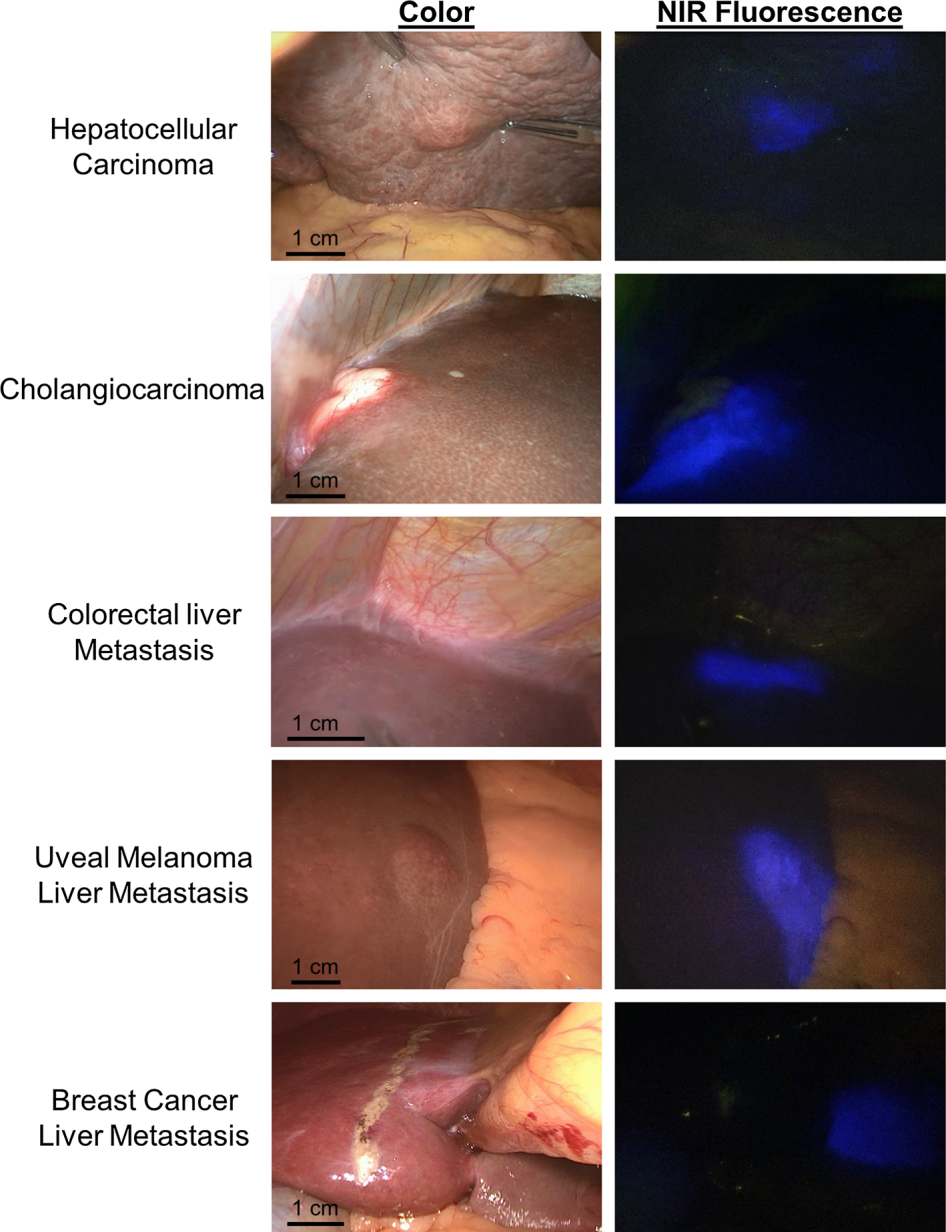
Representative images of intraoperative NIR fluorescence identification of liver tumors. A well-differentiated hepatocellular carcinoma shows the uptake of ICG in and around the tumor. A cholangiocarcinoma shows a rim-type fluorescence, similar to hepatic metastasis of colorectal and breast cancer and uveal melanoma. As the breast cancer and uveal melanoma liver metastases are located below the liver surface, the fluorescence does not show a rim. However, ex vivo a distinctive rim-type fluorescence signal is visible (data not shown). Of note, the fluorescence laparoscope does not possess an overlay function; the images are therefore not always aligned

No adverse events occurred due to ICG administration. Two patients (suffering from UMLM and CRLM) were excluded, because their procedure was postponed 3 and 6 days after ICG administration due to fever and logistical reasons. This delay resulted in intraoperative fluorescence signals too weak for sufficient guidance. In all other patients, fluorescence imaging of all types of tumors was successful (Fig. 1).

**Fig. 2:**
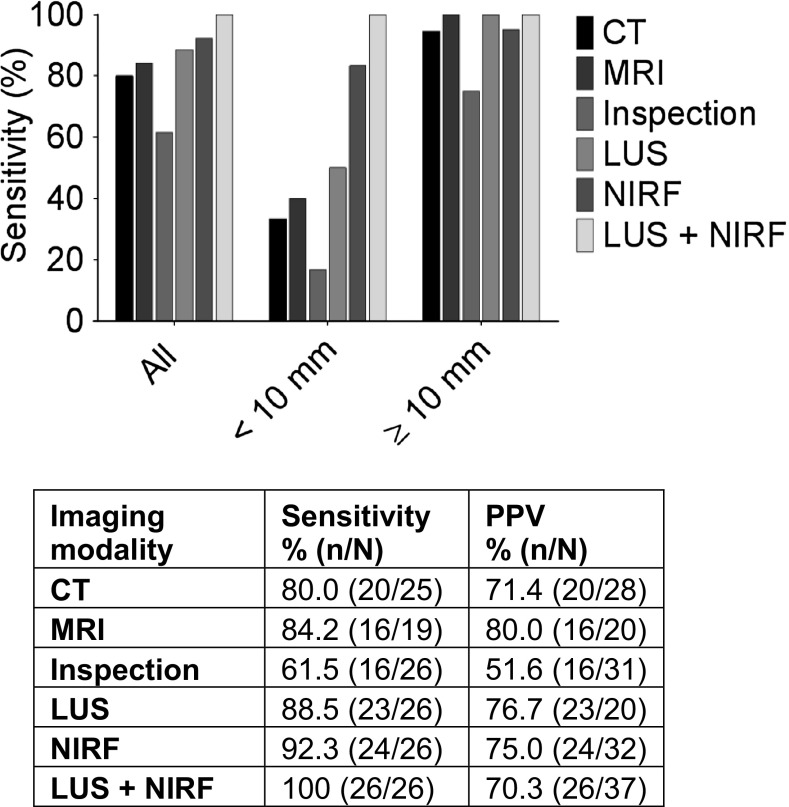
Sensitivity of all imaging modalities employed. Sensitivity and positive predictive value of computed tomography (CT), magnetic resonance imaging (MRI), visual inspection, laparoscopic ultrasonography (LUS), near-infrared fluorescence imaging (NIRF), and combination of LUS and NIRF. NIRF has the highest sensitivity rate among all imaging modalities. Combination of NIRF + LUS results in the detection of all hepatic tumors (100 % detection). The *graph* shows the sensitivity for all lesions together and divided into <10 and ≥10 mm. Sensitivity of all imaging modalities drops considerably for the detection of lesions <10 mm. However, all small lesions could still be detected by combining NIRF and LUS. Differences are not statistically significant

**Fig. 3:**
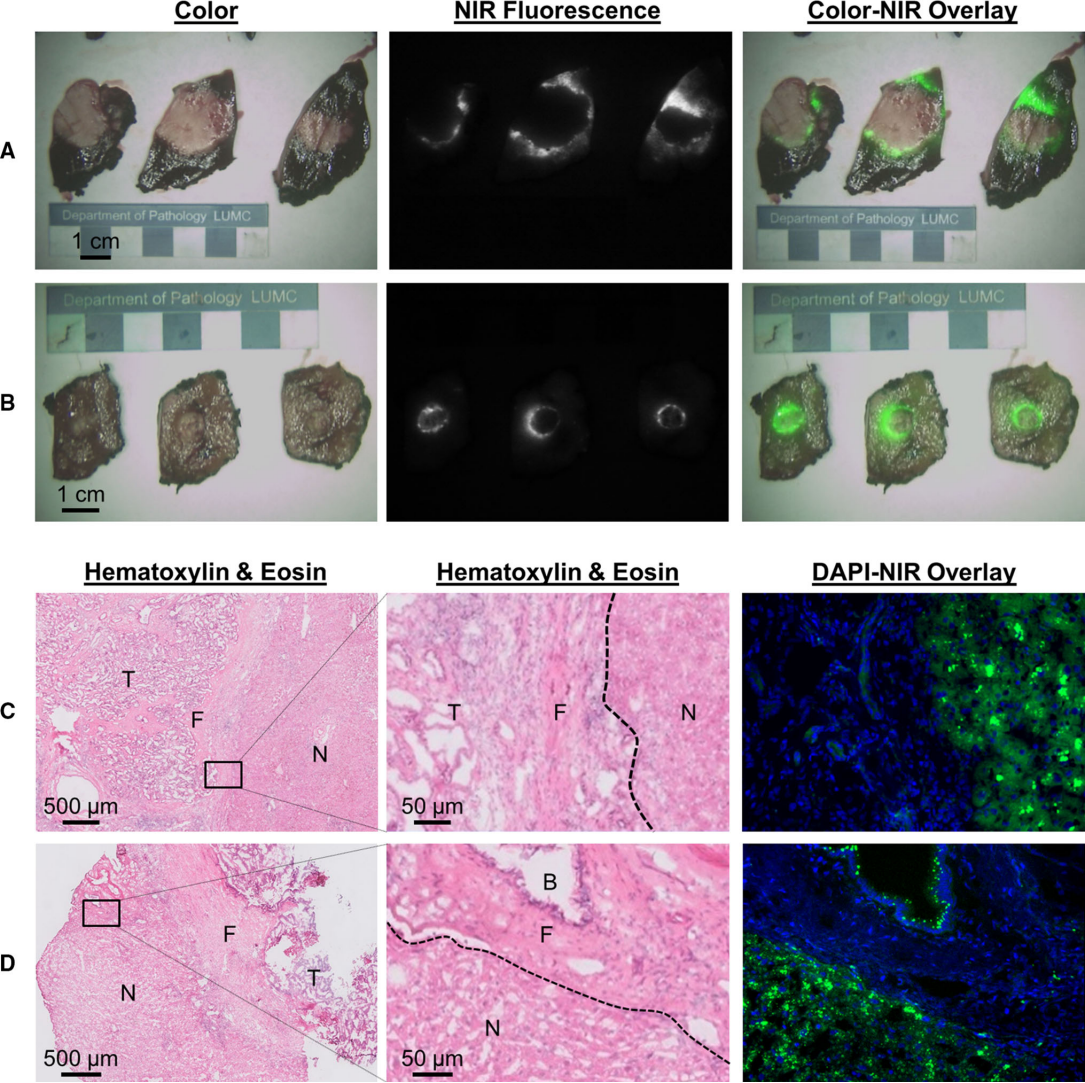
Ex vivo fluorescence imaging of resected tumors. Rim-type fluorescence surrounding a cholangiocarcinoma (ChC, **A**) and a colorectal liver metastasis (CRLM, **B**) imaged using a FLARE^®^ prototype. The CRLM was not visible using fluorescence imaging during surgery, because it was located >8 mm below the liver surface. Matching microscopic images (magnification ×2 and ×40) of hematoxylin and eosin and DAPI staining sections were made of ChC (**C**) and CRLM (**D**). Fluorescence shows a sharp demarcation between normal liver tissue and fibrosis or tumor tissue. **D** Also shows fluorescence in a biliary duct, probably due to mechanic obstruction by the tumor. *Abbreviations* *T* tumor, *F* fibrosis, *N* normal liver tissue, *B* biliary duct

A total of 46 lesions, including 26 malignant tumors with a median size of 20 mm, were resected in the remaining 20 patients (Table 2). Sensitivity and positive predictive values of the imaging modalities are shown in Fig. 2. Of all resected malignancies, 20 % were missed by CT scan, 16 % by MRI scan, 38 % by inspection, 12 % by LUS, and 8 % by NIR fluorescence imaging. Sensitivity of all imaging modalities of lesions sized <10 mm dropped considerably. However, these differences were not statistically significant. The lesions missed by LUS were all superficially located. The lesions missed by intraoperative NIR fluorescence imaging (a CRLM and UMLM) were located >8 mm below the liver capsule. During gross examination at the pathology department, the resected CRLM did show rim-type fluorescence (Fig. 3). The other false-negative lesion was located several centimeters below the liver capsule, and diagnosis was confirmed after biopsy. A sensitivity of 100 % was reached when fluorescence imaging and LUS were combined.

**Table 2:** Characteristics of resected lesions

Histopathological diagnosis	*N*	Size (mm)	Method of detection, *n*/*N* ^a^					
			CT	MRI	Insp	LUS	NIRF	LUS + NIRF
Malignant								
HCC	3	45 [25–58]	3/3	1/1	2/3	3/3	3/3	3/3
ChC	2	32 [30–33]	1/1	1/1	2/2	2/2	2/2	2/2
CRLM	18	18 [1–35]	14/18	11/14	10/18	15/18	17/18	18/18
UMLM	2	16 [14–18]	2/2	2/2	1/2	2/2	1/2	2/2
BCLM	1	11	0/1	1/1	1/1	1/1	1/1	1/1
Total	26	20 [1–58]	20/25	16/19	16/26	23/26	24/26	26/26
Benign								
Adenoma	1	20	1/1	–	1/1	1/1	0/1	1/1
FNH	1	84	1/1	–	1/1	1/1	1/1	1/1
Biliary adenofibroma	1	18	1/1	–	1/1	1/1	1/1	1/1
Bile duct hamartoma	6	4 [2–15]	1/6	1/5	5/6	1/5	1/6	2/6
Necrosis	4	13 [8–37]	2/4	2/4	3/4	2/4	3/4	3/4
No lesion identified^b^	2	–	1/2	1/2	1/2	0/2	0/2	0/2
Other^c^	5	–	1/3	0/4	3/5	1/5	2/5	3/5
Total	20	13 [2–84]	8/18	4/15	15/20	7/19	8/20	11/20

*Abbreviations* *Insp.* inspection, *HCC* hepatocellular carcinoma, *ChC* intrahepatic cholangiocarcinoma, *CRLM* colorectal liver metastasis, *UMLM* uveal melanoma liver metastasis, *BCLM* breast cancer liver metastasis, *FNH* focal nodular hyperplasia

^a^Not all lesions were imaged by all imaging modalities

^b^After gross examination, the pathologist could not identify any abnormal lesion in two specimens

^c^Includes cholestasis, cirrhotic tissue, fibrosis, and steatosis

No additional malignancies were identified only by intraoperative inspection. LUS alone identified one additional malignant tumor (Fig. 3B). NIR fluorescence imaging solely identified three additional malignancies in two patients suffering from CRLM. The first patient presented with a synchronous, solitary CRLM in segment 6, which partially responded to neoadjuvant chemotherapy. During laparoscopic metastasectomy, two additional lesions in segment 3 were visualized by typical rim-type fluorescence, whereupon resection of these lesions was performed (see Video 1 in ESM). Histopathological analysis confirmed the diagnosis of two metastatic intestinal-type adenocarcinomas of 1 and 3 mm, respectively. The second patient suffered from multiple CRLM resected by bi-segmentectomy. The additional occult lesion, sized 2 mm, was identified within the planned resection area.

**Fig. 4:**
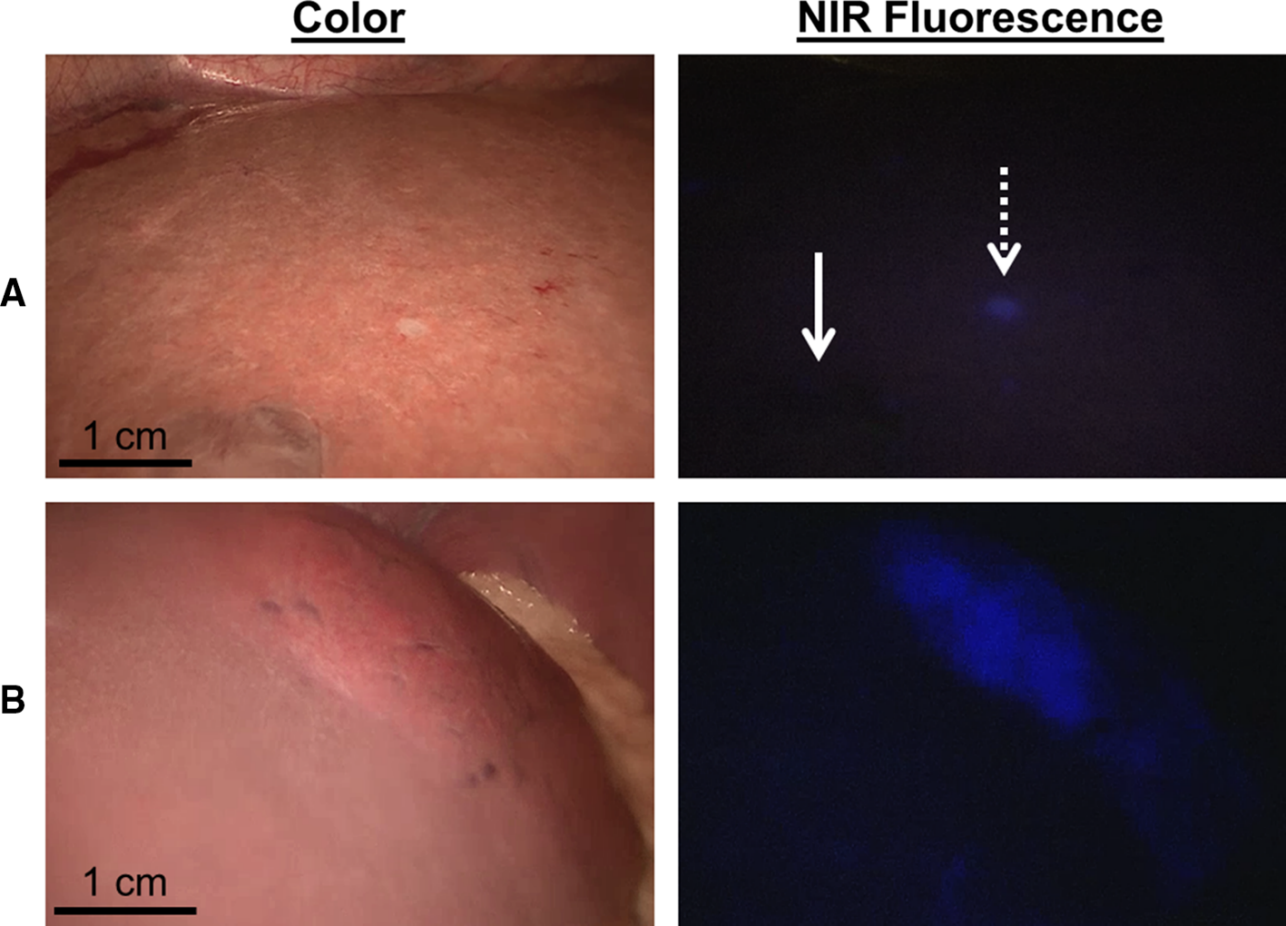
Fluorescence imaging of benign lesions. **A** Intraoperative imaging of a cyst (*white arrow*) and a bile duct hamartoma (*dashed arrow*). The cyst is not visible using fluorescence imaging, but the bile duct hamartoma is. The weak fluorescence signal does not show a distinctive rim-type fluorescence and can thereby be discriminated from a malignancy. **B** Benign, large focal nodular hyperplasia also shows NIR fluorescence. The mechanism of ICG uptake is unknown, but potentially its biliary excretion is disturbed

Positive predictive values were 71 % (CT), 80 % (MRI), 52 % (inspection), 77 % (LUS), 75 % (NIR fluorescence imaging), and 70 % (LUS combined with NIR fluorescence imaging). The differences did not reach statistical significance. Lesions that appeared suspicious by NIR fluorescence imaging included a bile duct hamartoma, steatotic liver tissue, necrosis (3 times), cholestatic and inflamed liver tissue, FNH, and a biliary adenofibroma (Fig. 4). Other benign lesions, such as cysts and fibrosis, were negative for NIR fluorescence. In hindsight, four benign lesions were unnecessarily removed because inspection alone was positive. One benign lesion was removed because NIR fluorescence imaging alone was positive.

Fluorescence imaging resulted in clear intraoperative demarcation of tumors. Resection could be performed more accurately using fluorescence guidance (see Video 2 in ESM). Prior to resection, the planned margins could be checked again by LUS. Histopathological evaluation of all resected malignant tumors revealed a radical resection rate of 88 % (22/25). One large UMLM was only biopsied to confirm diagnosis, since multiple similar liver lesions were detected during surgery. In one patient, intraoperative NIR fluorescence imaging after resection showed a fluorescent margin, located on the right portal vein. LUS and inspection did not show any remaining tumor. Based on the latter results, and the goal of preserving as much liver volume as possible, it was decided not to extend the resection to a full hemihepatectomy. Ex vivo evaluation of the resected tissue showed a tumor-positive resection margin, suggesting that a full hemihepatectomy should have been considered.

**Fig. 5:**
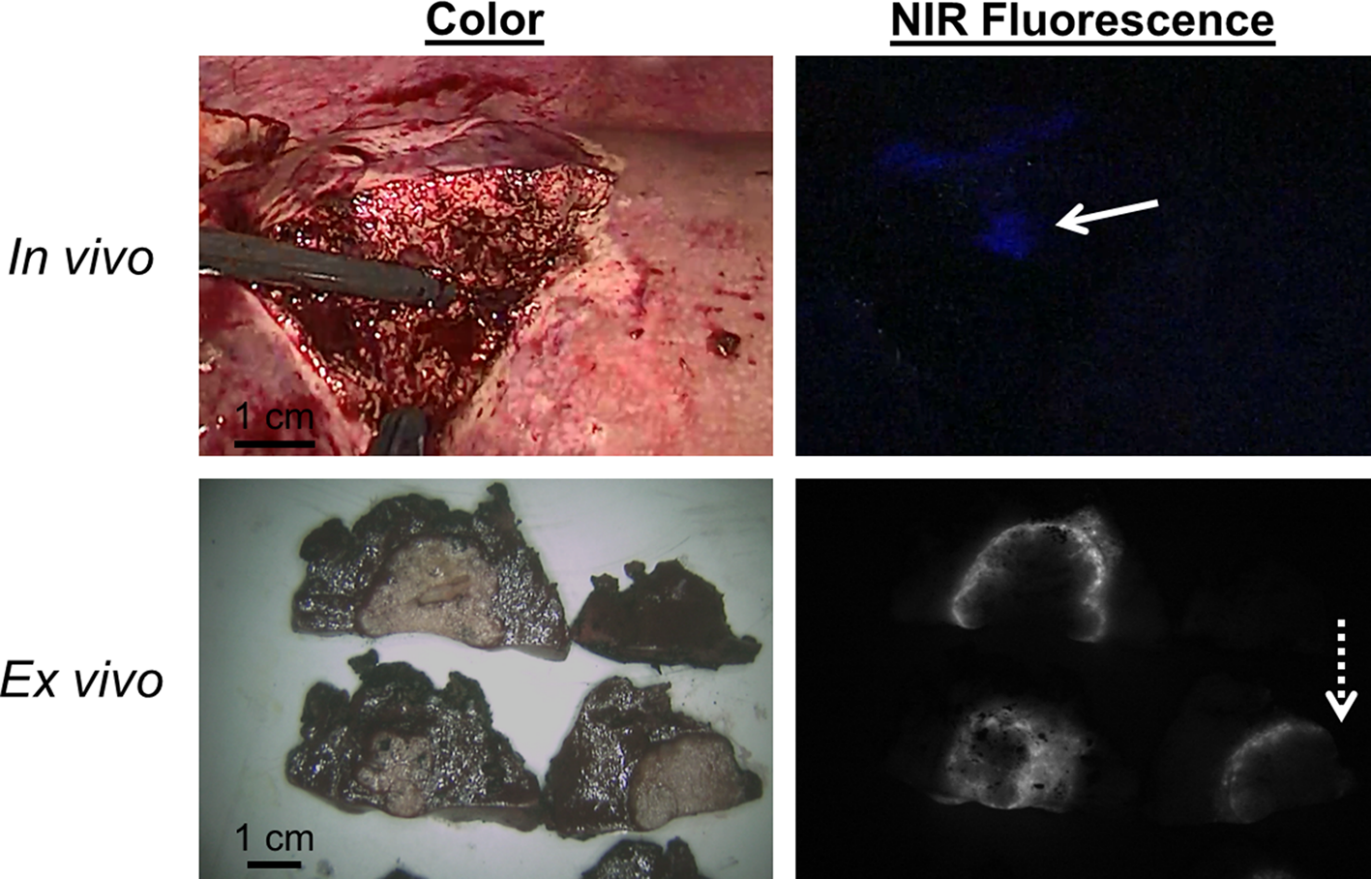
Intraoperative visualization of a positive resection margin of a colorectal liver metastasis. In vivo fluorescence is visible in the resection margin (*white arrow*), indicating a tumor-free margin of <8 mm. Ex vivo, the distinctive fluorescent rim is interrupted (*dashed arrow*) at the positive resection margin

In the second patient, the positive resection margin was in retrospect visible during surgery (Fig. 5). The positive resection margin in the third patient, suffering from a multinodular HCC, was not visible during surgery.

## Discussion

The present study describes the largest series of intraoperative fluorescence imaging during minimally invasive oncological liver surgery. Laparoscopic resection of liver tumors is believed to have several advantages over open surgery: less intraoperative blood loss, lower morbidity rates, and decreased length of hospital stay [12, 13]. Importantly, overall and disease-free survival remains similar for patients with HCC and CRLM. However, implementation of the laparoscopic technique occurs slowly. In France for instance, currently approximately 15 % of all liver resections are performed laparoscopically [14]. The main concerns include technical feasibility, safety of the technique, and the substantial learning curve [15]. Indeed, depth perception and the range of motion are hampered by a lack of tactile feedback and three-dimensional vision, as well as by altered orientation and scale of the surgical field [16]. No randomized controlled trials exist, but one can imagine that, compared to open procedures, the more technically demanding laparoscopic approach may expose patients to a higher risk of R1 resection. A recent meta-analysis showing a significant lower incidence of R1 resections after laparoscopic resection of CRLM may be the result of a selection bias, as some tumor locations with a higher risk of incomplete resection, such as the posterior segments, make an open approach preferable [17]. NIR fluorescence guidance during laparoscopic oncologic liver surgery reveals the localization of the tumor, enhances demarcation, and hopefully results in fewer incomplete resections. The current study showed that no NIR fluorescence should be visible at the resection margin to ensure a surgical margin of at least 8 mm. The radical resection rate was 88 % (22 of 25 malignancies) which is comparable with the literature [18]. All three incompletely removed tumors were located in the technically more demanding segments 6, 7, and 8.

Two patients from the current study were excluded because fluorescence imaging several days after ICG injection resulted in fluorescence signals that were too low. Other studies described adequate fluorescence imaging up to 2 weeks after ICG administration [19]. Patients included in these studies underwent a standard-of-care ICG clearance test to assess liver function and received a dose of 0.5 mg/kg ICG. In the Netherlands, this test is not standard of care. Moreover, we have previously optimized dose and timing of administration resulting in a lower dose by a factor of 3–5 [8]. The two excluded patients demonstrate that if a procedure is postponed, adequate fluorescence imaging demands a repeated dose of ICG, a higher initial dose of ICG, or a more sensitive fluorescence imaging system.

The appearance of the fluorescence signal during surgery depends not only on ICG dose and timing of administration, but also on tumor type and localization. Superficial metastases are recognizable by rim-type fluorescence, while deeper located metastases are completely surrounded by liver tissue and thus show total staining. In the current study, ex vivo fluorescence imaging showed rim-type fluorescence of all liver metastases and of the intrahepatic cholangiocarcinoma, while HCC also showed partial fluorescence. After intravenous administration, ICG is rapidly eliminated from the blood via the liver with a half-life of 2–3 min. Retention of ICG around liver tumors is not only due to simple mechanical compression of bile ducts. Recent studies revealed an underlying molecular mechanism. The transition zone between tumor and healthy liver tissue is composed of dedifferentiated hepatocytes with a keratine-7-positive immature phenotype [8]. These hepatocytes show down-regulation of anion transporters and are consequently unable to transport ICG into biliary canaliculi [20]. ICG is thus entrapped around tumors in these immature hepatocytes. Normally differentiated hepatocytes throughout the liver have already excreted their ICG and appear dark, just like metastases from extrahepatic tumors and poorly differentiated HCC, resulting in high contrast ratios. In differentiated HCC, the portal uptake function is preserved, leading to the accumulation of ICG in the tumor itself [21]. A similar mechanism may play a role in ICG retention in FNH and biliary adenofibroma, as shown in this study (Fig. 4). Observations in resected liver tumors showed that ICG is not only preserved in fresh tissue, but also in paraffin-embedded tissue [22]. Ex vivo fluorescence imaging remains possible in samples as old as 3 years.

The laparoscopic imaging system used in this study, i.e., the Karl Storz HD fluorescence laparoscope, allows easy switching between white light modus and fluorescence modus using a foot pedal. A drawback of this system is that no overlay of fluorescence and white light images is available. This makes orientation during fluorescence imaging sometimes challenging. Several other laparoscopic fluorescence imaging systems are available, including the commercially available Pinpoint endoscopic fluorescence imaging system (Novadaq Technologies Inc, Toronto, Canada) [23] and FireFly fluorescence imaging for the da Vinci Si surgical robot (Intuitive Surgical, Inc., Sunnyvale, CA, USA) [24]. These two systems do possess an overlay function, which may improve ease of use.

Near-infrared light (700–900 nm) has the advantage of higher photon penetration into tissue and lower autofluorescence compared to the visible light spectrum (400–700 nm). The reported depth penetration is however limited to a maximum of ≈8 mm [25]. In the present study, both lesions missed by fluorescence imaging were located >8 mm below the liver surface. Although the missed CRLM did show rim-type fluorescence ex vivo (Fig. 3B), these lesions were intraoperatively only visible using LUS. This finding demonstrates that fluorescence imaging must be seen as a complementary technique to LUS. The combination of LUS and fluorescence imaging reached a sensitivity of 100 %. However, only suspect lesions were resected, and this value is therefore not accurate, just like specificity and negative predictive value. No significant differences were found because this study was not designed and powered to do so. Deeper located liver tumors may also be visualized using intraoperative photoacoustic tomography [26]. This technique combines the specific uptake of ICG by some liver tumors with enhanced depth penetration of ultrasonography. Although feasible, technical improvements are required to make this technique available for clinical use.

Multiple studies illustrated the highly sensitive intraoperative detection of HCC and CRLM using fluorescence imaging [7, 8, 10, 21]. However, only one small study and three case reports describe this technique during minimally invasive liver surgery [9, 27–29]. Our group previously reported the feasibility of UMLM detection in three patients during laparoscopic resection [9]. Additional metastases were identified in two patients. Kudo et al. showed fluorescent detection of 12 out of 16 HCCs and 11 out of 16 CRLM in 17 patients [27]. Contrary to our current study, no comparison was made between lesions detected by fluorescence imaging and by other imaging modalities. Moreover, no information was provided about false-positive lesions detected by fluorescence imaging. The rather disappointing results of this study may be the result of a less sensitive imaging system, a suboptimal ICG dose, or variations in timing of ICG administration. To improve homogeneity, we chose to use a fixed ICG dose and to administer the dose the day before surgery. This strategy allowed visualization of all included tumors in vivo or ex vivo.

Fluorescence imaging can assist in differentiating between benign and malignant lesions, such as cysts, fibrosis, and hemangiomas. In the current study, four necrotic lesions were suspicious using fluorescence imaging. These lesions were resected in two CRLM patients who received neoadjuvant therapy. It may be that these lesions were in fact tumors that fully responded to the chemotherapy. In addition, two other resected lesions (a biliary adenofibroma and FNH) were considered false-positive, because they were benign. Nonetheless, imaging of these large lesions (18–84 mm) was still relevant as these lesions were preoperatively planned to be resected. Other studies describing fluorescence imaging during laparoscopic resection of liver tumors do not mention false-positivity rates.

Three occult, otherwise undetected, liver tumors (12 %) were identified in two patients suffering from CRLM. Other studies describe similar results. van der Vorst et al. [8] identified additional lesions in 12.5 % (5 of 40) of patients suffering from CRLM. Ishizawa et al. [21] examined resected liver specimens of patients with HCC and identified 7.7 % (21 of 273) additional HCCs that were not identified by gross examination. A key unanswered question is whether these additional lesions are “harbingers” of even more tumors not yet visible by any means or the only sites of disease that would otherwise have not been resected. One patient in the current study developed multiple liver metastases 3 months after surgery. The second patient is still disease-free after 6 months. Of the five patients with additional detected tumors in the study of van der Vorst et al. [8], two developed hepatic recurrence within 1 year, but three are currently still disease-free after a period of 3–6 years. Although these results should be interpreted cautiously, it appears that NIR fluorescence imaging can indeed prevent recurrence in some patients.

This study demonstrates the added value of intraoperative navigation using NIR fluorescence imaging during laparoscopic resection of a broad variety of malignant liver tumors. It is an easy, effective, and safe method. NIR fluorescence imaging can assist in localization and demarcation of known tumors, as well as identification of otherwise undetectable occult liver tumors. Large studies are required to confirm whether fluorescence-guided resection of liver tumors can improve radical resection rates and patient outcomes, but based on our results, we believe this method could quickly be an important component of laparoscopic resection of liver tumors.

## Electronic supplementary material

Below is the link to the electronic supplementary material.

Supplementary material 1 (WMV 121385 kb)

Supplementary material 2 (WMV 155111 kb)
